# Molecular Subtypes in Stage II-III Colon Cancer Defined by Genomic Instability: Early Recurrence-Risk Associated with a High Copy-Number Variation and Loss of *RUNX3* and *CDKN2A*

**DOI:** 10.1371/journal.pone.0122391

**Published:** 2015-04-16

**Authors:** Marianne Berg, Oddmund Nordgaard, Hartwig Kørner, Satu Oltedal, Rune Smaaland, Jon Arne Søreide, Kjetil Søreide

**Affiliations:** 1 Centre of Organelle Research (CORE), University of Stavanger, Stavanger, Norway; 2 Department of Gastrointestinal Surgery, Stavanger University Hospital, Stavanger, Norway; 3 Department of Hematology and Oncology, Stavanger University Hospital, Stavanger, Norway; 4 Department of Clinical Medicine, University of Bergen, Bergen, Norway; Institut national de la santé et de la recherche médicale, FRANCE

## Abstract

**Objective:**

We sought to investigate various molecular subtypes defined by genomic instability that may be related to early death and recurrence in colon cancer.

**Methods:**

We sought to investigate various molecular subtypes defined by instability at microsatellites (MSI), changes in methylation patterns (CpG island methylator phenotype, CIMP) or copy number variation (CNV) in 8 genes. Stage II-III colon cancers (n = 64) were investigated by methylation-specific multiplex ligated probe amplification (MS-MLPA). Correlation of CNV, CIMP and MSI, with mutations in KRAS and BRAFV600E were assessed for overlap in molecular subtypes and early recurrence risk by uni- and multivariate regression.

**Results:**

The CIMP phenotype occurred in 34% (22/64) and MSI in 27% (16/60) of the tumors, with noted CIMP/MSI overlap. Among the molecular subtypes, a high CNV phenotype had an associated odds ratio (OR) for recurrence of 3.2 (95% CI 1.1-9.3; P = 0.026). Losses of CACNA1G (OR of 2.9, 95% CI 1.4-6.0; P = 0.001), IGF2 (OR of 4.3, 95% CI 1.1-15.8; P = 0.007), CDKN2A (p16) (OR of 2.0, 95% CI 1.1-3.6; P = 0.024), and RUNX3 (OR of 3.4, 95% CI 1.3-8.7; P = 0.002) were associated with early recurrence, while MSI, CIMP, KRAS or BRAF V600E mutations were not. The CNV was significantly higher in deceased patients (CNV in 6 of 8) compared to survivors (CNV in 3 of 8). Only stage and loss of RUNX3 and CDKN2A were significant in the multivariable risk-model for early recurrence.

**Conclusions:**

A high copy number variation phenotype is a strong predictor of early recurrence and death, and may indicate a dose-dependent relationship between genetic instability and outcome. Loss of tumor suppressors RUNX3 and CDKN2A were related to recurrence-risk and warrants further investigation.

## Introduction

Colorectal cancer (CRC) is a major global health burden, and develops through the accumulation of genetic and epigenetic changes [1–3]. Genetic instability drives the process from neoplastic formation to invasive growth and development of metastasis. Thus, identifying specific molecular changes and their relationship to clinical endpoints (disease progress, recurrence or, death) may yield better understanding of the disease process. In CRC, three phenotypically different subgroups have been defined through instability in chromosomes, microsatellites or epigenetic alterations [3–5].

Copy number variation (CNV) refers to structural and numerical changes on the chromosome level, while microsatellite instability (MSI) occurs when repetitive base pair units have different number of repetitions in tumor cells compared to corresponding normal cells, which may produce a shift in the reading frame on the DNA. Last is the CpG island methylator phenotype (CIMP), which denotes an aberrant methylation spectrum of the DNA (hypo- or hypermethylation) that alters gene expression without directly involving genetic modifications [6–8].

Genetic alterations may involve large structural aberrations at the chromosomal level, and/or numerical changes at critical regions that can result in tumor promoting gene expression. For the majority of cancers, extensive chromosomal instabilities (CIN) are observed either as whole chromosome copy number change through gains and/or losses of chromosome regions, or as structural changes creating fusion genes [9–11]. Contrary to chromosomal and microsatellite changes or DNA mutations, epigenetic change does not alter the DNA itself but involves chemical modifications of the DNA such as methylation and histone modifications [12]. Gene expression is regulated through various mechanisms, including CpG-island methylation, in which increased methylation on the cytosines in the CpG sites reduces gene expression.

Historically, MSI and CNV (CIN) were regarded as mutually exclusive. However, more recently an overlap between the mentioned phenotypes has been shown [13]. Furthermore, the CIMP phenotype seems to be associated with the MSI phenotype, but combinations of all phenotypes including “triple negatives” have been reported [5]. MSI and BRAF mutation status is mainly observed in the same samples, and both are suggested to be clinically relevant [14,15]. Patients with tumors of the MSI type are associated with specific clinical, molecular and histopathological features [4,16–19], and have a better prognosis compared to patients with CNV tumors [20]. Furthermore, the particular tumor location along the colorectal continuum is associated with site-specific differences in the genetic composition of tumors. For example, rectal cancers are more prone to CNV type changes and colon cancers (in particular the proximal part) are more prone to the MSI phenotype [17]. Examination of current staging of CRC, which is largely based on the lymph node status[21], has recognized shortcomings and discrepancies that are debated [22]. Consequently, several investigators propose alternative molecular staging strategies [5,23–26], in which groups are based on the presence of CIMP, MSI, KRAS and BRAF mutations. While this has yet to reach clinical practice, the notion that sub-sites within the colon may harbor molecular differences that relate to distinct characteristics is of importance [17]. Previously we have demonstrated that lymph node numbers may be dependent on sub site location and their related molecular changes, which may be related to clinical outcome [19,27]. Thus, further exploration of the genomic differences in node-negative and node-positive colon cancer is of interest to enhance understanding of CRC and related disease behavior.

Copy number variants (CNVs) are recognized as an important type of genetic variation that modifies human phenotypes [28,29], including human cancers. Multiplex ligation-dependent probe amplification (MLPA) is an increasingly common used technique for determining relative DNA sequence dosage (or copy number variation) [29]. MLPA is a multiplex PCR assay that utilizes up to 40 probes that are each specific for a different DNA sequence (mainly exons of a specific gene of interest), to evaluate the relative copy number of each DNA sequence [29]. MLPA can also be used for methylation status determination, copy number analysis in segmentally duplicated regions, expression profiling, and transgene genotyping. MLPA is a cost-efficient alternative over more labor-intensive and costly methods, such as array comparative genomic hybridization (aCGH) [29,30]. Moreover, MLPA allows for simultaneous investigation of methylation status and CNV in tumors, such as previously reported in retinoblastoma [31].

Thus, the aim of this study was to explore the prevalence of molecular subtypes in stage II and III colon cancer patients using MLPA to determine the methylation pattern and CNVs in a defined probe set, and to evaluate its relationship with aggressive disease behavior, defined as early recurrence (<3 years) after surgery.

## Materials and Methods

### Patient samples

The study cohort is derived from a consecutive series of 213 stage I-III surgically treated colon cancers at Stavanger University Hospital during a 4 year period, with details as described previously [32]. The study was approved by the regional ethics committee (#197.04) and all patients gave informed consent prior to inclusion. All patients underwent surgery with curative intent. Patients with lymph node positive (pN+) stage III disease who were physiologically fit (e.g ECOG performance status 0–1), were offered adjuvant therapy according to national guidelines at the time.

For the current project, a sub-cohort of patients with colon cancer stage II and III were recruited. Patients who had either a follow-up of at least 36 months at the time of selection (= 3 years, by which time >90% of recurrences occur) or who had died of CRC at commencement of the current project were eligible. A total of 64 patients fulfilled these criteria and were included for MLPA analysis.

Follow up was performed using the 11-digit social security number and access to electronic patient files for any sign of recurrent disease or a fatal event related to colon cancer. The selection was based on the information available at 3 years follow up, thus excluding any patient with shorter follow-up at commencement of the study. Recurrence was defined as any locoregional or systemic relapse of the disease at time of follow up.

### DNA extraction

DNA from the 64 patients’ tumor samples was extracted and isolated from fresh frozen tissue using DNeasy Mini kit or AllPrep DNA&RNA Mini Kit (Qiagen, Hilden, Germany), as described previously [33].

### Multiplex ligation-dependent probe amplification (MLPA)

The multiplex ligation-dependent probe amplification procedure, using the SALSA MS-MLPA kit ME042-B1 CIMP probe mix (for detecting methylation status in the promoter regions of 8 different genes; RUNX3, MLH1, NEUROG1, CDKN2A, IGF2, CRABP1, SOCS1 and CACNA1G) as given in Table 1, were performed according to the vendors recommendations (MRC-Holland, Amsterdam, the Netherlands), and as previously described [34]. As an activating mutation of BRAF^V600E^ is tightly associated with CIMP positivity [35], a mutation specific probe is included in the SALSA MS-MLPA kit that detects the V600E (1799T>A) somatic mutation if this is present in the sample.

**Table 1:** Sequences, chromosomal location, and fragment lengths for the investigated positions/probes in the MLPA method.

**Gene name**	**Fragment length**	**Chromosome location**	**Mapview** ^§^	**5' probe sequence**	**3' probe sequence**
RUNX3	346	01p36.11	01–025.128720	CCGGTGGACGTGCTGGCGGACCACGCA	GGCGAGCTCGTGCGCACCGACAGCCCCAACTTCCTCT
RUNX3	372	01p36.11	01–025.128920	CCGCTTGGGTCTACGGGAATACGCAT	AACAGCGGCCGTCAGGGCGCCGGGCAGGCGGA
RUNX3	256	01p36.11	01–025.129597	GCTAGAAATTTGCTTAGAACGTCCGGGTC	CCACGGAAGGCGCCCTTGCCGCCCTCTCT
MLH1	355	03p22.2	03–037.009360	TCCGCCACATACCGCTCGTAGTAT	TCGTGCTCAGCCTCGTAGTGGCGCCTGACGTCGCGTT
MLH1	463	03p22.2	03–037.009621	CTGCTGAGGTGATCTGGCGCAGA	GCGGAGGAGGTGCTTGGCGCTTCTCAGGCTCCTCCTCT
MLH1	132	03p22.2	03–037.009760	CAAGAGCGGACAGCGATCTCTAACGCGCAA	GCGCATATCCTTCTAGGTAGCGGGCAGTAGCCGCTTCAGG
MLH1	177	03p22.2	03–037.010228	GACACGCCTCTTTGCCCGGGCAGA	GGCATGTACAGCGCATGCCCACAACGGCGGAGGC
NEUROG1	166	05q31.1	05–134.898938	TGCGTCCAGGGCCGCGTTCAA	GTTGTGCATGCGGTTGCGCTCGCGATCGTTGGCCTTG
NEUROG1	282	05q31.1	05–134.899244	GTGTCCGTCGGTCCTGCACAGCGCAAC	GATGCCAGCCCGCCTTGAGACCTGCATCTCCGACCTC
NEUROG1	211	05q31.1	05–134.899351	GGCCGCCAGGGCGCACTTACGT	TCCCAACAGCCTGGGGTTGTTACTCTGTGCCAGTTGCGGG
NEUROG1	389	05q31.1	05–134.899479	CTGATCTGATCGCCGGCGACATCA	CTCAGGAGACCGGCCGGGCGCGTGGCCC
NEUROG1	364	05q31.1	05–134.899537	CCCATTGTTGCGCCGGGTACTTA	AGGGGTCCTGAGGCCAGTCGTGTGCCACACTCGGTGCT
NEUROG1	202	05q31.1	05–134.899663	CCTCATCCCCGTGCAGCGCCCGGGTATTTGCATAAT	TTATGCTCGCGGGAGGCCGCCATCGCCCCTC
BRAF	409	07q34	07–140.099560	CCTTTACTTACTACACCTCAGATATATTTCTTCATGAAG	GAAATCTCGATGGAGTGGGTCCCATCAGTTTGAACAGTTGTCTGG
CDKN2A	232	09p21.3	09–021.964677	CACCTGGATCGGCCTCCGACCGTAAC	TATTCGGTGCGTTGGGCAGCGCCCCCGCCTCCAGCAGC
CDKN2A	183	09p21.3	09–021.965200	CTTTTAACAGAGTGAACGCACTCAAACACGCCTTTGCT	GGCAGGCGGGGGAGCGCGGCTGGGAGCAGGGAGGC
CDKN2A	335	09p21.3	09–021.984268	GCAGGTTCTTGGTGACCCTCCGGA	TTCGGCGCGCGTGCGGCCCGCCGCGAGTGAG
CDKN2A	195	09p21.3	09–021.985276	GGAAGAGGAAAGAGGAAGAAGCGCTCAGAT	GCTCCGCGGCTGTCGTGAAGGTTAAAACCGAAAATAAAAATGG
IGF2	171	11p15.5	11–002.117594	TCAAGCCACCTGCATCTGCACTCA	GACGGGGCGCACCCGCAGTGCAGCCTCC
IGF2	418	11p15.5	11–002.118681	CCACCGCCTGCCACAGAGCGTTCGATCGC	TCGCTGCCTGAGCTCCTGGTGCGCCCGCGGAC
IGF2	141	11p15.5	11–002.118895	GAAATTTCTCTCTAGCGTTGCCCAAACACA	CTTGGGTCGGCCGCGCGCCCTCAGGACGTGG
CRABP1	207	15q25.1	15–076.419820	GCCACCATGCCCAACTTCGCCGGCAC	CTGGAAGATGCGCAGCAGCGAGAATTTCGACGAGCTGC
CRABP1	310	15q25.1	15–076.420033	GCTGAACGCGTGGGTTCCGGGATCTCT	ACCAGCTTCTCCGAGACCCGGTGCGCCTGGGAGACAA
CRABP1	265	15q25.1	15–076.420493	GTGGAGATCCGCCAGGACGGGGATCAG	TTCTACATCAAGACATCCACCACGGTGCGCACCACTG
CRABP1	319	15q25.1	15–076.420701	CCTTTGCAGCCTGTGGCGCGCCTTCCT	TGCAGGGTGTGTACACTGGCTGTTTGCAGAGGGGGTTTGTGCATCCTAG
SOCS1	239	16p13.13	16–011.256544	CCGATTCTACTGGGGGCCCCTGAGCGTGCACG	GGGCGCACGAGCGGCTGCGCGCCGAGCCCGT
SOCS1	154	16p13.13	16–011.256960	GACTTGGTGCTCCGTGCTCGCCCCCT	AGGGCCGGGTCCGCCGGGAGCGCCGCCCT
SOCS1	399	16p13.13	16–011.257200	CCTTTCTCCGGCCCTAGCCCAAATCGCCCA	GACCAGGCGCGGATCCCAGCCTGGCCAGCAGGCGGCG
SOCS1	300	16p13.13	16–011.257552	CCAGCCCCGCCTCCGAGCCGGTTTAAA	AGACTGGCGCAGGGGCGGGCGCCGAACAGAGCGA
CACNA1G	273	17q21.33	17–045.993509	GAGCCTGGGCGCGAAGCGAAGAA	GCCGGAACAAAGTGAGGGGGAGCCGGCCGGC
CACNA1G	246	17q21.33	17–045.993744	CGGGCGATCCGGAGAGGGGCA	AGCGGCGCCCCTCAGAGGAGGTGTCCTCACGCAA
CACNA1G	218	17q21.33	17–045.993972	GCGGCTGTCCTGGCTCAAGTAGAAGAA	AACCACCGGGGCCAGCGCCGGGTACGGC

^§^Refers to hg18

From all 64 DNA samples, 100 ng DNA was heat-denatured in a total volume of 5 μl Tris-EDTA buffer, and further performed as recommended by the vendor. Briefly, a mixture of probe-mix (ME042, MRC-Holland, Amsterdam, the Netherlands) and buffer were added to the denatured DNA, and probes were allowed to hybridize to the DNA at 60C for 16 hours. Each sample was divided into two tubes: one of which was ligated, while the other was ligated and then digested using the methylation-sensitive restriction enzyme *HhaI*. Both reactions were then subjected to a PCR reaction using a thermal cycler (GeneAmp 2700, Applied Biosystems, Foster City, CA, USA), and fragment analysis performed on a capillary sequencer (ABI 3130*xl*, Applied Biosystems, Foster City, CA, USA).

Extracted DNA from normal colonic mucosa was used as a normal reference (n = 12 samples). Additionally, the colorectal cancer commercial cell-lines Caco-2 and HT-29 were used as cancer controls (see supporting information, S1 Data file, and S1 and S2 Figs).

The raw data from the analysis were analyzed using Coffalyser.NET (beta version, MRC-Holland, Amsterdam, the Netherlands).

### Definitions

#### CIMP

Criteria for scoring of CIMP phenotype were more than 20% methylation of a minimum of one third of the probes within at least three of the five genes in the Weisenberger panel[35], as previously described [34].

#### CNV

For CNV phenotype scoring, all eight genes investigated were used. Copy number variation (CNV) was used as a proxy for chromosomal instability (CIN) in the study. Loss and gain were defined using the relative ratio of sample vs reference for a probe were less than 0.7, or higher than 1.3, respectively. For region analysis, aberration in one or more probes was defined as CNV of the region, and a high CNV phenotype if five or more of the eight regions (1p36.11, 3p22.2, 5q31.1, 9p21.3, 11p15.5, 15q24.2, 16p13.13 and 17q21.33) were aberrant.

#### MSI

The MSI phenotype was investigated based on the Bethesda panel of genes using fragment analysis, as previously described [36]. Furthermore, methylation status of the MLH1 gene using MLPA was used to correlate the methylation status in relation to MSI. In the following, MSI status refers to the results from the Bethesda panel, whereas MLH1 investigated by the MLPA method is referred to as MLH1 methylation.

### Mutational analyses

KRAS mutational analyses were performed as previously described in [37]. BRAF mutation status was determined based on two different methods. One, the MLPA method [34], in which the BRAF mutation specific probe generated a signal if the V600E mutation was present, and; second, by using conventional PCR and sequencing analysis, as described previously [38].

## Ethics

Ethical approval for the study was obtained from the Regional Ethics Committee, and all patients consented to inclusion in the study, as previously described [19].

## Statistical analysis

All statistical analyses were performed using the *Statistical Package for Social Sciences* (IBM SPSS v. 20). Descriptive data are presented as numbers and rates (%) or medians with interquartile ranges (IQR), if not otherwise stated. Mann-Whitney U-test was used to compare continuous data between groups when data did not have a normal distribution. Fisher’s exact test was used for 2x2 tables to compare categorical data, and odds ratio (OR) presented with 95% confidence interval (95% CI) for significant risk factors. A multiple logistic regression analysis using both enter and forward modeling was performed to evaluate independent risk-factors for early recurrence (yes/no) as a dichotomous variable. Gender and age were included in the model, and models were controlled for CIMP, CNV and MSI as well as KRAS, BRAF mutation status. Significant factors found on univariate risk-analyses were included in the prediction model. A Hosmer-Lemeshow Goodness-of-fit test was performed to indicate stability of the model [39], and contribution to the model variation evaluated by R-square statistics for logistic regression analysis. All tests were two-sided and a P<0.050 was considered statistically significant.

## Results

### Clinical information on recurrence and survival

Of the 64 patients, 58% were females, and median age was 76 years at time of surgery. Clinicopathological and molecular data for the included subjects are presented in Table 2. Early recurrences were observed in 26 patients (41%), of which 16 had systemic recurrence, 7 had loco-regional recurrence, and three had both loco-regional and systemic recurrences. Among the 26 patients with recurrent disease, 15 (58%) had died of the disease (23% of the study cohort) at the time of study inclusion. As expected, 66% of stage III (node positive) patients experienced recurrence compared to 23% in stage II, for an OR of 6.9 (95% CI 2.2–21.3; p<0.001).

**Table 2:** Clinicopathological and molecular features in the patient samples series.

**Clinicopathological and molecular features**	**All patients N = 64 (100%)**	**Alive >3 years N = 49 (77%)**	**Died of disease N = 15 (23%)**	**Univariate log. Reg,OR [95% CI]**	**P-value, Chi-square/ Fisher exact test** ^#^
Gender					
Female	37 (58)	28 (57)	9 (60)	1.13 [0.35–3.65]	0.845
Male	27 (42)	21 (43)	6 (40)		
Age; years, median (range)	76 (30–92)	76.0 (30–92)	77.0 (63–91)	-	
Stage					
II	40 (63)	37 (76)	3 (20)	12.3 [2.97–51.17]	<0.001
III	24 (38)	12 (25)	12 (80)		
Tumor localization in colon					
Right	41 (64)	29 (59)	12 (80)	0.36 [0.09–1.45]	0.220
Left	23 (36)	20 (41)	3 (20)		
Tumor size					
< 5 cm in diameter	24 (38)	20 (41)	4 (27)	0.53 [0.15–1.89]	0.377
≥ 5 cm in diameter	40 (63)	29 (59)	11 (73)		
Recurrence					
Present	26 (41)	38 (78)	0 (0)	-	-
Absent	38 (59)	11 (22)	15 (100)		
Adjuvant treatment					
No	50 (78)	42 (86)	8 (62)	5.3 [1.44–19.11]	0.008
Yes	14 (22)	7 (14)	7 (38)		
KRAS status					
wild type	43 (67)	31 (63)	12 (80)	0.43 [0.11–1.73]	0.348
mutated	21 (33)	18 (37)	3 (20)		
BRAF status					
wild type	49 (77)	39 (80)	10 (67)	0.6 [0.12–3.10]	0.542
mutated	15 (23)	10 (20)	5 (33)		
^*^MSI status					
MSS	44 (73)	33 (72)	11 (79)	0.69 [0.16–2.89]	0.740
MSI	16 (27)	13 (28)	3 (21)		
MLH1 methylation status					
Unmeth	46 (72)	16 (33)	2 (13)	3.15 [0.63–15.67]	0.198
Meth	18 (28)	33 (67)	13 (87)		
CIMP status					
negative	42 (66)	32 (65)	10 (67)	0.94 [0.28–3.2]	0.923
positive	22 (34)	17 (35)	5 (33)		
CNV status					
CNV low (= chromosomal stable)	39 (61)	33 (67)	6 (40)	3.1 [0.94–10.20]	0.057
CNV high (= CIN)	25 (39)	16 (33)	9 (60)		

*MSI status missing in 4 included patients

^#^Fisher exact test if less than five cases in one of the groups

Numbers subject to rounding.

### Microsatellite instability and mutational data

The MLPA analyses from tumor samples were technically successful in all 64 included patients, with regards to the methylation analysis, the chromosomal analysis, and the BRAF^V600E^ mutation analysis. In addition, the KRAS mutation analyses were successful for all 64 patients, and 60 (94%) had a successful MSI status.

**Fig 1:**
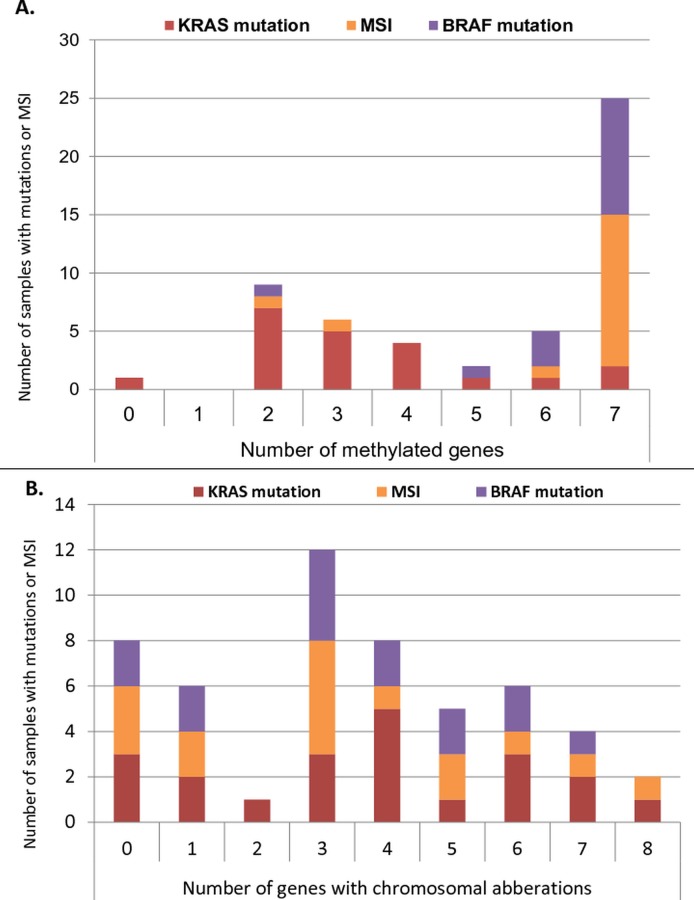
Distribution of KRAS and BRAF mutations and microsatellite instability. (A) Distribution vs number of methylated genes (none had methylation in all genes, thus 8 not displayed). (B) Distribution vs the number of genes with chromosomal aberrations

The frequency of mutations in KRAS, BRAF^V600E^ and presence of MSI per the number of methylated genes or per number of chromosomal aberrations is presented in Fig 1A and 1B, respectively.

MSI was found in 16 (27%) of the samples. Methylation of MLH1 was detected in 17 (28%) samples, and methylation of MLH1 and MSI was concomitantly observed in 14 (23%) of the patients (p<0.001, Fishers exact test). The MSI and CIMP phenotype, as well as the BRAF^V600E^ mutation, and MLH1 methylation were significantly statistically associated with right-sided tumor location (p<0.01, p<0.01, p<0.01 and p = 0.01 for MSI, CIMP, BRAF^V600E^ and MLH1 meth, respectively, using Fishers exact test).

The median number of methylated genes differed significantly between MSI and MSS (microsatellite stable) samples, (7 and 2, respectively; p<0.01), BRAF wild-type and mutated samples (2 and 7; p<0.01), larger tumor size and smaller than 5 cm (3 and 2; p = 0.04), and male and female sex (2 and 4; p = 0.03).

The BRAF^V600E^ mutation was observed in 15 (23%) samples, 11 within the MSI group, indicating a significant covariation of the two aberrations, (p<0.01). Furthermore, BRAF^V600E^ mutations were significantly associated female sex (p = 0.02), methylation of MLH1 (p<0.01), and CIMP phenotype (p<0.01). BRAF results were validated by demonstrating BRAF-wt in all (n = 12) normal samples and in the Caco-2 cell line, but with BRAF^V600E^ mutation present in the HT-29 cell line, as expected.

Mutations in KRAS were detected in 20 samples (33%), of which eight samples were negative for CNV, CIMP and MSI. Mutations in both KRAS and BRAF coexisted in two samples, and in three cases KRAS mutation and MSI were concomitantly observed.

### Genome complexity

**Fig 2:**
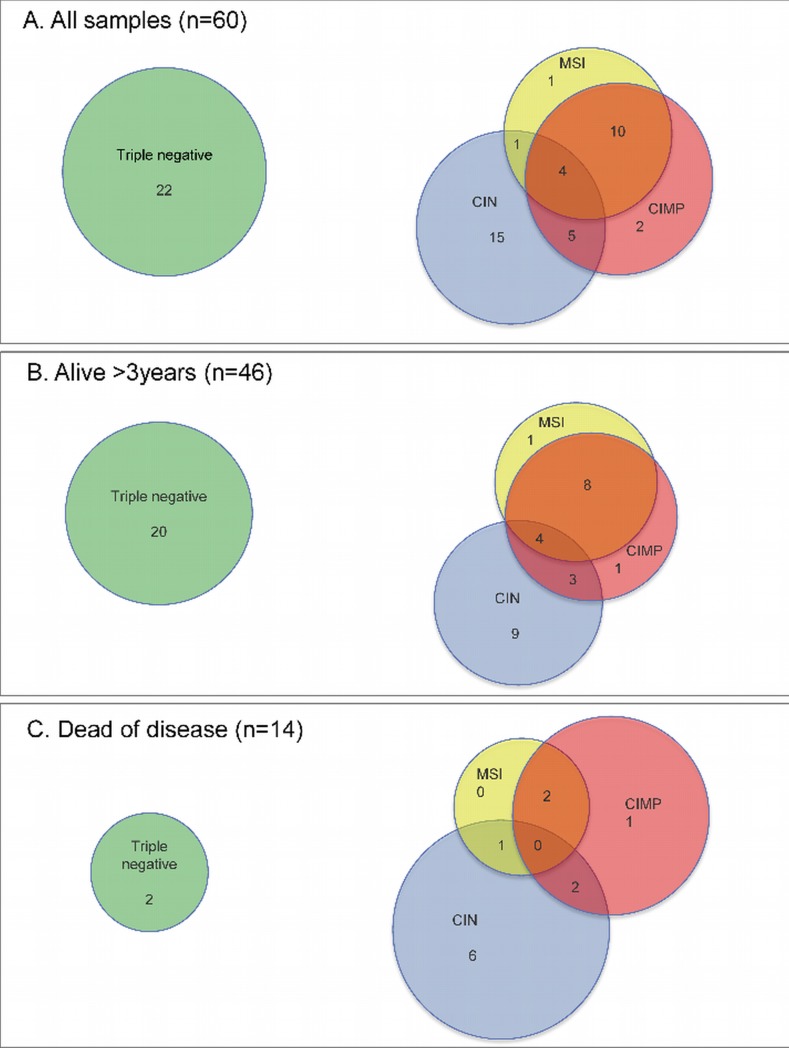
Venn diagrams illustrating the frequencies of the molecular phenotypes. (A) The total study population, (B) Patients alive with no recurrence (surviving for >3 years), (C) Patients with early recurrence or death (within 3 years after surgery).

The samples were categorized for all phenotypes, and grouped based on their combined molecular phenotypes. Overlaps between all phenotypic subgroups were observed. The combined tumor phenotypes for all patients deceased and living are presented in Table 3, and Fig 1A and 1B and Fig 2A–2C, respectively.

**Table 3:** Frequency of the different phenotypic subgroups.

**Phenotypes**	**Study population n = 64 (%)**	**Alive >3 years n = 49 (%)**	**Died of disease n = 15 (%)**
MSI only	1 (2)	1 (2)	0 (0)
CNV only	15 (23)	9 (18)	6 (40)
CIMP only	2 (3)	1 (2)	1 (7)
MSI+CNV	1 (2)	0 (0)	1 (7)
MSI+CIMP	10 (16)	8 (16)	2 (13)
CNV+CIMP	5 (8)	3 (6)	2 (13)
Triple negative	22 (34)	20 (41)	2 (13)
Triple positive	4 (6)	4 (8)	0 (0)
ND	4 (6)	3 (6)	1(7)

ND, denotes not dermined

Comparing frequencies of combined phenotypes showed statistically significant difference for the survivor group compared to the deceased (p = 0.037, binary logistic regression). Of note, the triple negative and the CNV only phenotype were different between the deceased patients and the survivors: 13% vs 41% and 40% vs 18%, respectively (Fig 2B and 2C). Isolated MSI and CNV coexisted in only one sample (2%), while 4 additional patients also had CIMP (triple positive, 6%). MSI and CIMP were present in the same samples in 10 (16%) cases, whereas CIMP and CNV were observed for five (8%) samples. The MSI and CIMP phenotypes most frequently presented in combination with other phenotypes, and as the single phenotype for only one (2%) and two (3%) cases, respectively. In contrast, the CNV phenotype existed as the sole phenotype for 15 (23%) of the samples.

Among the different phenotypes, the triple positive and the triple negative were the two groups displaying the fewest recurrences, 0/4 (0%) and 4/22 (18%), respectively. For the other phenotypic groups, the recurrences ranged from 40–100%. Furthermore, the frequency of triple negatives in the patient subgroup without recurrences (18/35) was found to be significantly different from the recurrence subgroup (4/25), p = 0.007.

### Aberrations and risk of early recurrence

For the CIMP phenotype, a statistically significant association was observed with female sex (p = 0.001), right-sided location of the tumor (p = 0.001), and in tumors displaying the MSI phenotype (p<0.01).

The high CNV phenotype was observed in 25 (39%) of 64 samples, and was associated with recurrent disease. High CNV phenotype was found in 14 of 25 (56%) recurrences, but only 11 of 39 (28%) of those alive, for an associated odds ratio of 3.2 (95% CI 1.1–9.3; P = 0.026).

Statistical significance was also observed in the number of chromosomally aberrant genes between deceased patients and survivors (CNV median 6, IQR 3–7 vs CNV median 3, IQR 1–5; P = 0.001),Fig 2A.

**Fig 3:**
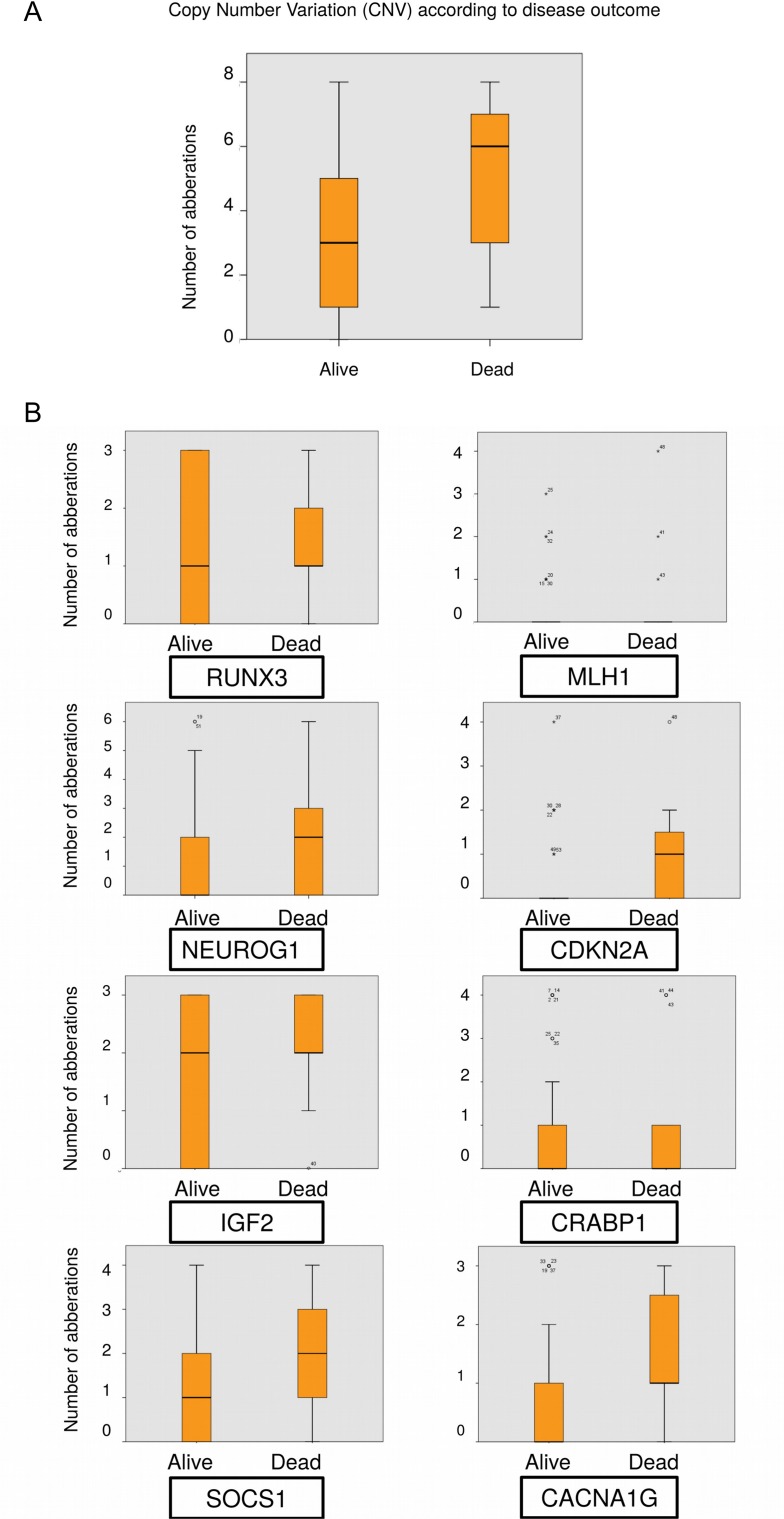
Copy number variation (CNV) in relation to clinical outcome. (A) Boxplot showing the number of aberrant genes in deceased patients (right) and survivors (left). (B) Boxplot showing the number of aberrant chromosome positions in each of the genes studied, in recurrent (or deceased) patients (right) and patients with no early recurrence (left).

**Fig 4:**
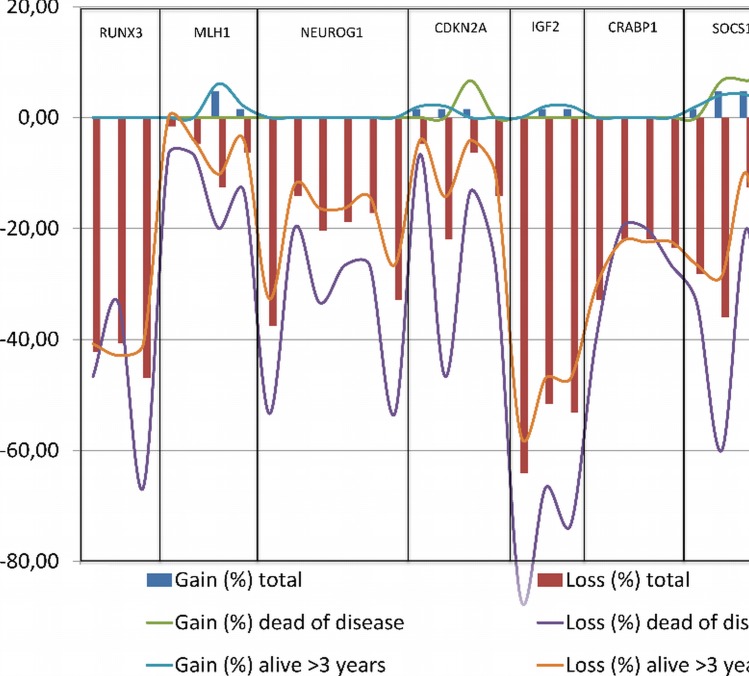
Bar chart illustrating the percentage of aberrant patient samples per probe. Red bars represent lost regions and blue bars represent gains. Lines illustrating gains and losses in patients alive >3 years (blue and purple, respectively), and patients deceased of disease (green and red, respectively)

There were more aberrations in the early recurrence group overall when separately comparing the number of aberrant probes for each gene between patients with no recurrence and those with early recurrence, Fig 3. The difference in number of aberrant positions between survivors and deceased for CDKN2A, IGF2 and CACNA1G was statistically significant, Fig 4B. When using the dichotomized status (aberrant/normal) for each gene, aberrations in CACNA1G (p = 0.007), CDKN2A (p = 0.003) and RUNX3 (p = 0.032) were significantly associated with disease related death.

More specifically, loss of CACNA1G conferred an OR of 2.9 (95% CI 1.4–6.0; P = 0.001) for recurrence, as 18 of 30 (60%) of patients with this CNV recurred compared to only 7 of 34 (21%) with normal status. Loss of IGF2 was associated with an OR of 4.3 (95% CI 1.1–15.8; P = 0.007), as 23 of 47 (49%) recurred, compared to 2 of 17 (12%) with normal status. Loss of CDKN2A had an OR of 2.0 (95% CI 1.1–3.6; P = 0.024), with 61% recurrence for those with loss, compared to 30% for those with normal status. Finally, loss of RUNX3 was associated with an OR of 3.4 (95% CI 1.3–8.7; P = 0.002) with recurrence occurring in 64% with loss compared to 16% of those with normal status.

There were no significant difference between stage II and III regarding the frequency of the three different molecular phenotypes (MSI, CNV and CIMP), and the combined phenotypes, as well as BRAF and KRAS mutations. Inactivation of CACNA1G due to methylation was observed most frequently in BRAF mutated and MSI tumors (p≤0.01), and most frequently in tumors located in the proximal colon.

### Multivariable modeling of early-recurrence risk

In a multiple logistic regression model with the variables CNV, CIMP, MSI, KRAS, BRAF, stage, tumor localization and gender, the only variable that was retained in the model for recurrence risk was stage. Analyzing cancer-death as an outcome using the same variables, CNV status was included together with stage in the model, but with wide confidence intervals for the adjusted ORs.

Introducing the independent gene aberrations significantly associated with early recurrence risk (losses of RUNX3, CDKNA2A, CACNA1G and IGF2) and controlling for the above mentioned molecular subtypes and age, gender, stage and tumor location, revealed a final model that included only RUNX3 and CDKN2A together with stage as predictors of early recurrence (Table 4). A Hosmer-Lemeshow Goodness of fit test (Chi-square 3.844; p = 0.572) indicated a robust model. The R-square statistics (Cox&Snell and Nagelkerke) was reported between 0.316 and 0.427, indicating that the model explained between 31.6% and 42.7% of variation in the risk estimation.

**Table 4:** Multivariable model for early risk of recurrence.

Factor		B	S.E.	Wald	Df	P	OR	OR 95% C.I. (Lower/Upper)
Step1^a^	Stage II vs III	1.764	.582	9.189	1	.002	5.833	1.865/18.245
	Constant	-.253	.291	.758	1	.384	.776	
Step2^b^	Stage II vs III	1.808	.634	8.133	1	.004	6.099	1.760/21.131
	Loss_RUNX3	1.770	.699	6.405	1	.011	5.871	1.491/23.126
	Constant	-.550	.345	2.538	1	.111	.577	
Step 3^c^	Stage II vs III	2.013	.692	8.467	1	.004	7.489	1.929/29.067
	Loss_CDKN2A	1.622	.738	4.831	1	.028	5.063	1.192/21.510
	Loss_RUNX3	1.801	.725	6.175	1	.013	6.053	1.463/25.045
	Constant	-.214	.384	.310	1	.577	.808	

Variables in the Equation

^a^ Variable(s) entered on step 1: Stage_II_III.

^b^ Variable(s) entered on step 2: Loss_RUNX3.

^c^ Variable(s) entered on step 3: Loss_CDKN2A.

## Discussion

In the current study, we investigated the interaction and overlap between molecular subtypes in colon cancer with development of early recurrence after surgery. As described, there is overlap between several molecular features that complicates the clear distinction between groups for molecular and clinical relevance. Using MLPA for a panel of 8 genes, we demonstrated that the gene dosage effect of methylation and chromosomal aberrations is in part associated with molecular signature and presence of mutations, and is also related to risk of early recurrence. In addition to stage III, early recurrence was associated with loss of RUNX3 and CDKN2A, both of which are known tumor suppressor genes in CRC and previously reported to be associated with clinical outcome in CRC [40–43]. This may potentially be used as prognostic information in addition to the current TNM-staging system to avoid misclassification, as lymph node status (definition of node negative or positive disease) has several shortcomings, which has been discussed in detail elsewhere [21,22]. Also, defining patients at risk for early recurrence may facilitate targeted and better tailored surveillance after surgery[44].

For colorectal cancer, three broad molecular phenotypes are described, including CNV, MSI and epigenetic changes [3,7,8]. Investigators have proposed distinct classification of 3, 4 and 5 groups in the past [14,24–26], but none has yet reached clinical practice. The extent of overlap between these phenotypic groups has been investigated [5,14,16,25,45–47], but the clinical implications of the molecular phenotypes are so far vague. The use of different methodology, definitions, scoring criteria, and differences in the type of patient material investigated make comparisons difficult, which is underscored by the overlap found in this study. Additionally, and as we have documented recently, the use of different criteria for CIMP-status may differs with the use of included genes and probes [34], and thus generates different results between studies, which hinders comparison.

In total, MSI was observed in 27% of the colon cancers. This is in line with other studies in which rectal cancers are not included, as colon cancers have a higher prevalence of MSI. Both the MSI phenotype and MLH1 methylation were found in 14/60 samples (23%). Two samples displayed the MSI phenotype without MLH1 methylation, and three samples showed MLH1 methylation without MSI. The extensive overlap between these features supports the fact that MLH1 inactivation causes MSI. However, a discrepancy in MLH1 methylation and MSI has also been observed by others [48,49], and illustrates that several DNA mismatch repair genes including MLH1 cause MSI. On the contrary, methylation of MLH1 does not necessarily lead to impairment of the mismatch repair machinery.

The BRAF^V600E^ mutation status was predicted using both conventional DNA sequencing and MLPA with specific probes for the point mutation. Both results were in 100% concordance, and show the MLPA methodology to be reliable.

Mutations in BRAF^V600E^ and KRAS were originally reported as mutually exclusive [50]. A double activation of the MAPK pathway has been suggested to lead to differentiation and cell senescence, rather than growth promotion as is the result of a single mutation [51]. However, for two patients in our sample series, the mutations were found to coexist, albeit with numbers too low to allow for any clinical interpretation.

Patients with early recurrence showed numerically more chromosomal aberrations (Figs 3 and 4). This supports the suggestion that CNV could predict a worse prognosis [52], as indicated by a high CNV in the current study. For stage II patients, the numerical difference in chromosomal aberrations between recurrence and no recurrence was statistically significant (p = 0.022), as was the numerical difference for deceased or surviving patients (p = 0.007). This difference could not be observed for stage III patients. As stage II patients are normally treated with surgery alone, this could be used as a prognostic marker for recurrence, and potentially as an aid when selecting stage II patients who would benefit from adjuvant therapy. However, the results should be interpreted with caution based on the sample size and validated in larger patient series to be confirmed.

One interesting observation is that chromosomal aberrations, rather than methylation itself, of genes included in the CIMP panel seems to be most important for clinical outcome of disease. This implies that the genes in the CIMP panel have tumor suppressor activity, and that their mechanistic function is important with regards to colon cancer, and not only for methylation. Indeed, both RUNX3 and CDKN2A (also known as p16) are known tumor suppressor genes. Aberrations in RUNX3 have been reported as an early event in colorectal cancer progression[40]. The normal activity of the CDKN2A (also known as p16) gene is as a tumor suppressor, preventing uncontrolled cell proliferation by initiating cell cycle arrest and apoptosis. The prognostic significance of CDKN2A inactivation in colorectal cancer has been studied, but no clear associations has been found [43]. Further, normal function of CACNA1G affects cell proliferation and apoptosis, and disturbing this calcium signaling might be important in cancer, as these processes guide further progress in cellular life. This indicates its instrumental importance, and that nonfunctional CACNA1G will switch the cells over to a more aggressive function [53]. Also, the protein hormone IGF2 is involved in development and growth of the cell, and is involved in carcinogenesis [54,55].

When investigating the number of methylated positions, there was significantly more methylation in MSI tumors compared to MSS, and in BRAF mutated samples compared to wild-type. This is most probably because methylation of CRC-critical genes causes MSI in non-familial colorectal cancer [56]. However, the number of methylated positions was also statistically significant larger in large tumors compared to smaller, and in tumors from females compared to males.

## Conclusions

Stage II-III colon cancer patients who experience early cancer recurrences after surgery had significantly more chromosomal aberrations (median 6 vs. median of 3 for living patients) than patients with no evidence of disease at 3 years follow up. Loss of the tumor suppressor genes RUNX3 and CDKN2A (p16) appear to hold important clinical information in addition to node-status (stage III; node positive disease). The additional role of CACNA1G and IGF2 warrants further studies, both to investigate tumor-regulating mechanisms and to confirm the clinical role in larger patient samples.

## Supporting Information

S1 Data Rawdata of obtained results on file (Excel). (XLSX)

S1 Fig Illustration of rawdata from copy number analyses of normal and control samples. Cut off for scoring of gain and loss is indicated.
(TIF)

S2 Fig Illustration of rawdata from methylation analyses of normal and control samples. Cut off for scoring of methylation is indicated.
(TIF)
