# Supplementary figures and images for: Molecular Subtypes in Stage II-III Colon Cancer Defined by Genomic Instability: Early Recurrence-Risk Associated with a High Copy-Number Variation and Loss of RUNX3 and CDKN2A
Source: PLoS One. 2015 Apr 16;10(4):e0122391. doi: 10.1371/journal.pone.0122391 (PMC4399912; doi:10.1371/journal.pone.0122391)

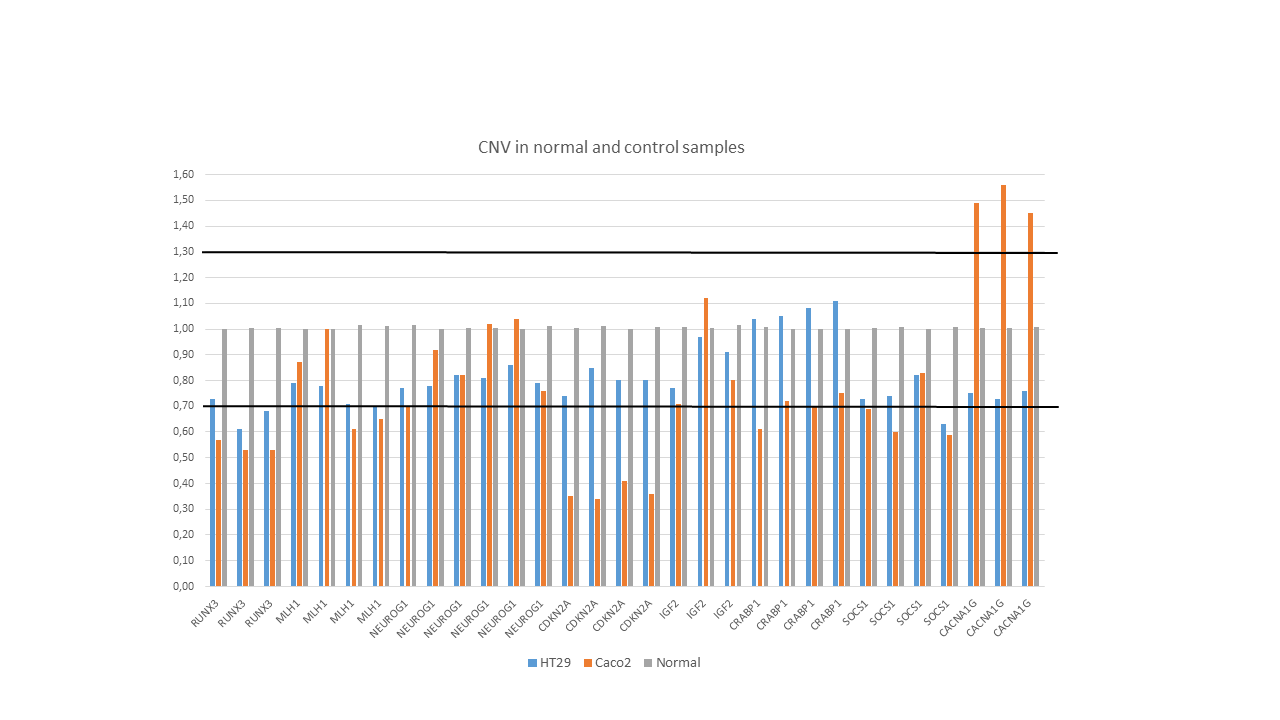

Supplement: S1 Fig — Cut off for scoring of gain and loss is indicated. (TIF) [file pone.0122391.s002.tif]

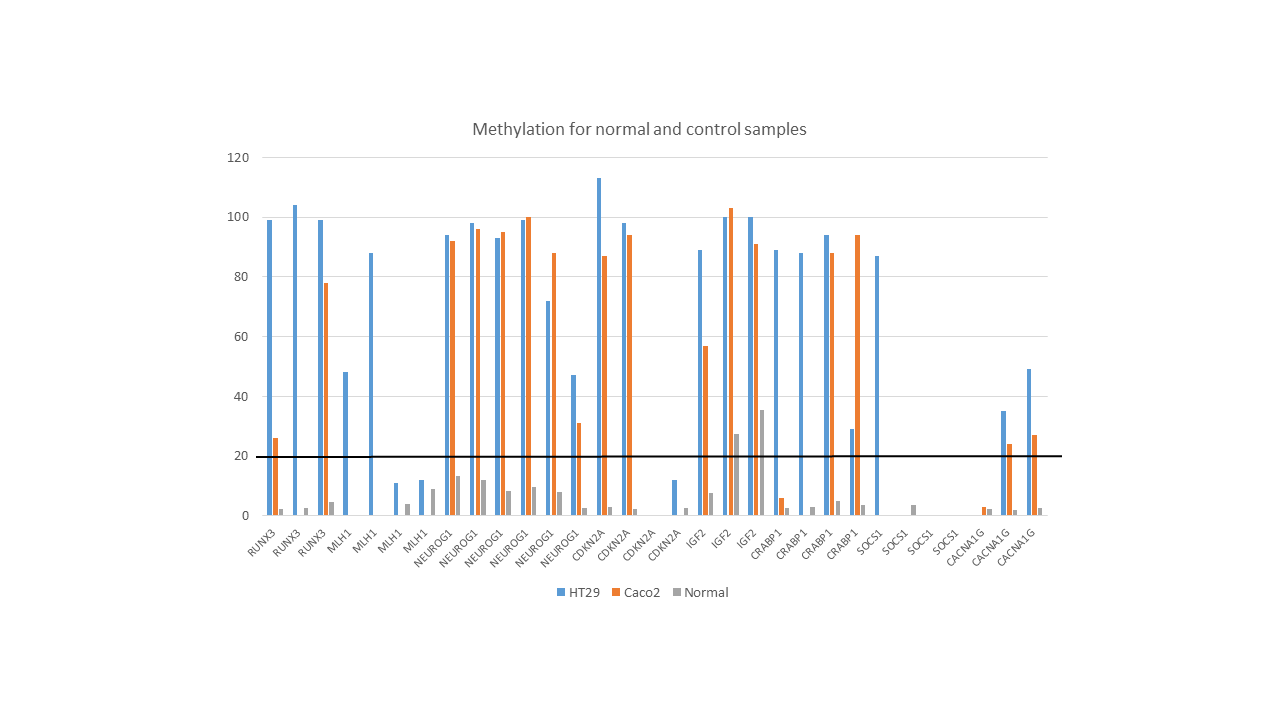

Supplement: S2 Fig — Cut off for scoring of methylation is indicated. (TIF) [file pone.0122391.s003.tif]
